# New Oleoyl Hybrids of Natural Antioxidants: Synthesis and In Vitro Evaluation as Inducers of Apoptosis in Colorectal Cancer Cells

**DOI:** 10.3390/antiox9111077

**Published:** 2020-11-03

**Authors:** Gabriele Carullo, Sarah Mazzotta, Adrian Koch, Kristin M. Hartmann, Oliver Friedrich, Daniel F. Gilbert, Margarita Vega-Holm, Regine Schneider-Stock, Francesca Aiello

**Affiliations:** 1 Department of Biotechnology, Chemistry and Pharmacy, Department of Excellence 2018-2022, University of Siena, Via Aldo Moro 2, 53100 Siena, Italy; gabriele.carullo@unisi.it; 2 Department of Pharmacy, Health and Nutritional Sciences, Department of Excellence 2018-2022, University of Calabria, Edificio Polifunzionale, 87036 Rende (CS), Italy; 3 Department of Pharmaceutical Sciences, University of Milan Via Luigi Mangiagalli 25, 20133 Milano, Italy; sarah.mazzotta@unimi.it; 4 Department of Organic and Medicinal Chemistry, Faculty of Pharmacy, University of Seville, Profesor García González 2, 41071 Seville, Spain; mvegaholm@us.es; 5 Institiute of Pathology, University Hospital, Friedrich-Alexander University Erlangen-Nürnberg Universitätsstr. 22, 91054 Erlangen, Germany; adrian.koch@uk-erlangen.de; 6 Experimental Tumorpathology, University Hospital, Friedrich-Alexander University Erlangen-Nürnberg Universitätsstr. 22, 91054 Erlangen, Germany; 7 Institute of Medical Biotechnology Friedrich-Alexander-University Erlangen-Nürnberg, Paul-Gordan-Str. 3, 91052 Erlangen, Germany; kristin.m.hartmann@fau.de (K.M.H.); oliver.friedrich@fau.de (O.F.); daniel.gilbert@fau.de (D.F.G.); 8 Erlangen Graduate School in Advanced Optical Technologies (SAOT), Friedrich-Alexander-University Erlangen-Nürnberg, Paul-Gordan-Str. 6, 91052 Erlangen, Germany

**Keywords:** quercetin, oleic acid, cytotoxicity, polyphenol hybrids, pancreatic porcine lipase, colorectal cancer, HCT-116, apoptosis, DNA damage

## Abstract

Nowadays, the beneficial role of a healthy lifestyle, particularly emphasizing the quality of foods and cancer management, is accepted worldwide. Polyphenols and oleic acid play a key role in this context, but are still scarcely used as anti-cancer agents due to their bio-accessibility limits. Therefore, we aimed to synthesize a set of new oleoyl-hybrids of quercetin, morin, pinocembrin, and catechin to overcome the low bioavailability of polyphenols, throughout a bio-catalytic approach using pancreatic porcine lipase as a catalyst. The in vitro assays, using a wide panel of human cancer cell lines showed, mainly for two novel regioisomer oleoyl-hybrids of quercetin, a remarkable increase in apoptotic cell populations. We suggested that the DNA damage shown as ɣH2AX signals might be the major cause of apoptotic cell death. Finally, we demonstrated convincing data about two novel polyphenol-based hybrids displaying a highly selective anti-cancer cytotoxicity and being superior compared to their reference/parental compounds.

## 1. Introduction

**Figure 1:**
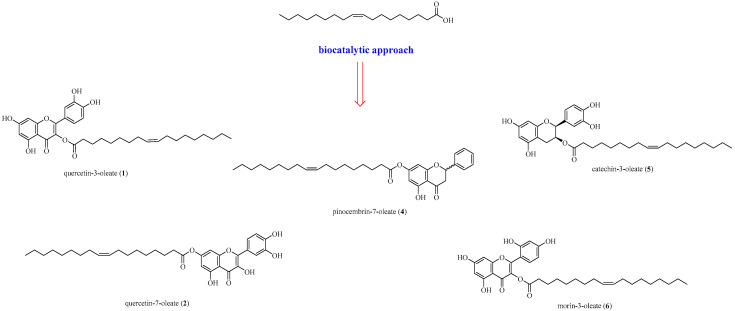
Design of new polyphenol hybrids as potential antiproliferative agents.

Colorectal cancer (CRC) is a multistep process involving several genetic or epigenetic changes culminating in the transformation of normal into malignant cells characterized mainly by the uncontrolled proliferation [1,2]. Despite advances in early detection techniques, modern surgery, and personalized chemotherapeutic treatments, the management of cancer is still a challenging clinical context due to metastasis, drug resistance, dose-limiting toxicity, and high degrees of relapse [3]. In this scenario, the development of new anticancer agents seems to be urgent. Natural compounds are promising sources for the development of new remedies for different diseases and it has been estimated that numerous types of cancer can be prevented by a healthy lifestyle [4]. Many studies established that polyphenolic compounds have positive influences on health, such as antidiabetic, antiviral, antioxidant, anti-inflammatory, and anticancer effects. Polyphenols showed strong in vivo and in vitro anti-proliferative effects on various human cancer cells with few or no toxicity effects on normal cells [5,6]. They are known to majorly target the p53 signalling pathway to produce an anticancer activity through apoptosis in a variety of cancers [7,8]. Furthermore, human epidemiological studies have exhibited the possible impact of dietary polyphenols on the prevention of many cancer diseases together with the use of olive oil [9]. From this point of view, polyphenols as plant derived secondary metabolites could become a useful alternative especially when co-administered with other drugs. It is well documented that phenolic compounds exert anticancer properties by reducing both intrinsic and extrinsic factors interfering with the metabolism of pro-carcinogens, for example, by regulating the expression of cytochrome P450 enzymes [10,11]. In particular, quercetin (QUE) is one of the most abundant flavonoids found in a variety of plant-based foods such as capers, red onions, red grapes, parsley, broccoli, lovage, and a number of berries, endowing a plethora of interesting biological activities [10,11]. QUE has been shown to exhibit an anticancer activity against different human cancer cell lines, including breast, colorectal, stomach, head and neck, lung, ovarian, melanoma, and leukemia through multiple mechanisms of action. Since no obvious toxic side effects on normal cells are known, there was a growing interest in its therapeutic effect on tumours [12,13]. In addition, its positional isomer morin, possesses comparable biological properties. Many reports highlighted the anticancer potential of this naturally occurring bioactive phytochemical [14,15]. Giving a glance to flavanones, pinocembrin showed several biological activities including anticancer, anti-inflammatory, antimicrobial, and antioxidant properties [16,17]. This flavanone is mainly present in honey and propolis [18,19], but it is also one of the characteristic secondary metabolites of the leaves of *Glycyrrhiza glabra L.* (licorice). Furthermore, catechin, especially (+)-catechin, the form present in tea leaves, has many beneficial properties for human health such as anticancer, anti-obesity, antidiabetic, cardiovascular, anti-infectious, hepatoprotective, and neuroprotective effects [20,21]. Another important natural compound is oleic acid (OLAC) available in high contents in extra virgin olive oil, the main fat used in the Mediterranean diet, to which amazing healthy properties are ascribed. Experimental and clinical findings suggest that olive oil has a protective effect in colorectal cancer patients [22,23]. Olive oil is a complex mixture of fatty acids, mainly OLAC, and minor compounds such as phenolic compounds, lignans, hydrocarbons, and triterpenes. OLAC is the endogenous ligand of free fatty acid receptor 1 (FFA1) and FFA4, implicated in the pathogenesis of various types of human cancers [24,25,26,27,28]. Despite the positive effects of these natural-occurring drugs, their poor bioavailability, and regarding OLAC, its lipotoxicity limits the therapeutic employment as nutraceuticals. Moreover, in many countries the Mediterranean diet it is not easily accessible and the preventive effects of these kinds of foods against cancer should have been lost. To overcome these strong limits, several hybrid compounds were designed and synthesized, using a bio-catalytic approach, oleoyl ester-based of QUE, morin, pinocembrin, and catechin to explore their antiproliferative activity in a wide panel of human cancer cell lines and to propose a possible mechanism of action (Figure 1).

## 2. Materials and Methods

### 2.1. General Chemistry Methods

Commercially available reagents (Merck products) were used without further purification, and all solvents were of HPLC grade. Reactions under nitrogen atmosphere were performed in oven- or flame-dried glassware and anhydrous solvents. All the reactions were monitored by thin-layer chromatography (TLC) Merck 60 F254 silica plates. Analytical TLC was conducted on Merck aluminum sheets covered with silica (C60). The plates were either visualized under UV-light (254 nm) or stained by dipping in a developing agent followed by heating [KMnO_4_ (3 g in water (300 mL) along with K_2_CO_3_ (20 g) and 5% aqueous NaOH (5 mL)]. Flash column chromatography was performed using silica gel 60 (0.040–0.063 mm). All the new compounds were characterized by ^1^H-NMR and ^13^C-NMR. For the recording of ^1^H-NMR and ^13^C-NMR, a Bruker Advance (operating at 300 MHz) was used. Unless otherwise stated, all NMR spectra were recorded at 25 °C. The chemical shifts (δ) are reported in parts per million (ppm) and the coupling constants (*J*) in Hz. For spectra recorded in CDCl_3_, signal positions were measured relative to the signal for CHCl_3_ (δ 7.26 ppm for ^1^H-NMR and 77.16 ppm for ^13^C-NMR). For spectra recorded in DMSO-*d_6_*, signal positions were measured relative to the signal for DMSO (δ 2.50 ppm for ^1^H-NMR and 39.52 ppm for ^13^C-NMR). For spectra recorded in Acetone-*d_6_*, signal positions were measured relative to the signal for Acetone (δ 2.60 ppm for ^1^H-NMR and 30.0 and 206 ppm for ^13^C-NMR). Quercetin anhydrous 99% (HPLC) powder, Morin hydrate, (+)-catechin hydrate ≥98% (HPLC), powder, oleic acid ≥99% (GC), EDCI, HOBt, pancreatic porcine lipase (PPL), acetic anhydride, imidazole were purchased from Merck (Italy). Pinocembrin was extracted as previously reported by us [19,29]. Elemental analyses were performed on the Leco Trunspec CHNS Micro elemental system. The purity of final compounds was evaluated by C, H, N analysis and it was confirmed to be ≥95%.

#### 2.1.1. Synthesis of 2-(3,4-diacetoxyphenyl)-4-oxo-4*H*-chromene-3,5,7-triyl triacetate (Intermediate **8a**)

Procedure a): To a solution of **8** (1.0 equiv) in acetic anhydride (20 equiv), 15 mL of pyridine was added. The solution was heated to reflux and stirred for 5 h. Fifty grams of ice-water was added to the warm mixture. The resulting precipitate was filtered and washed with cold ethyl acetate to afford **8a** as a white solid (79% yield). Spectroscopic data are in agreement with those reported.

#### 2.1.2. Synthesis of 4-(3,5-diacetoxy-7-hydroxy-4-oxo-4*H*-chromen-2-yl)-1,2-phenylene diacetate (Intermediate **8b**) 

Procedure b): In an ice/acetone bath (−15 °C) to a solution of **8a** (1.0 equiv) in DCM (10 mL), a solution of imidazole (2.0 equiv) in 5 mL of DCM was added dropwise. The resulting solution was allowed to warm to room temperature (rt) and stirred for 2 h. The reaction mixture was diluted in 50 mL of DCM and washed with 3N aqueous HCl (3 × 50 mL). The organic layer was then dried over anhydrous Na_2_SO_4_, filtered and evaporated under reduced pressure. The pure compound was obtained after flash-column chromatography of the residue (eluent: CHCl_3_/methanol, 97/3), giving **8b** as a white solid (87% yield). Spectroscopic data are in agreement with those reported.

#### 2.1.3. Synthesis of 2-(3,4-dihydroxyphenyl)-3,5-dihydroxy-4-oxo-4*H*-chromen-7-yl oleate (Compound **2**)

Procedure c): A solution of OLAC (1.0 equiv), **8b** (1.5 equiv), and HOBt (1.2 equiv) in 2.5 mL of dry DCM was cooled at 0 °C and stirred under N_2_ atmosphere for 15 min. Thereafter, a solution of CMC (1.5 equiv) in 3.5 mL of dry DCM was added dropwise. The mixture was stirred at rt for 24 h, then the solvent was washed twice with a 5% aqueous NaHCO_3_ solution, and once with brine. The collected organic layers, dried on anhydrous Na_2_SO_4_, were concentrated under reduced pressure. Without further purification, the crude compound (1.0 equiv) was added to a solution of CH_3_CN (20 mL) and 6N aqueous HCl (10 mL). The resulting solution was stirred and refluxed for 1.5 h. Then, 100 mL of ethyl acetate and 100 mL of water were added. The organic layer was washed with 3N aqueous HCl (3 × 100 mL), dried over anhydrous Na_2_SO_4_, and filtered. The solvent was evaporated under reduced pressure to obtain compound **2** as an olive-green resin (77% yield). ^1^H NMR (Acetone-*d_6_*): 12.15 (bs, 1H), 8.60 (bs, 1H), 8.36 (bs, 1H), 8.06–7.98 (m, 1H), 7.80–7.72 (m, 1H), 7.65 (d, 1H, *J* = 8.40 Hz), 6.93 (d, 1H, *J* = 8.40 Hz), 6.48–6.44 (m, 1H), 6.22–6.18 (m, 1H), 5.38–5.20 (m, 2H), 2.22 (t, 2H, *J* = 7.3 Hz), 2.05–1.89 (m, 4H), 1.60–1.42 (m, 2H), 1.39–1.11 (m, 20H), 0.85 (t, 3H, *J* = 8.0 Hz). ^13^C NMR (Acetone-*d_6_*): δ 175.6, 174.0, 164.0, 161.4, 161.3, 156.8, 147.4, 146.0, 144.9, 135.8, 129.7, 129.6 (2C), 129.5 (3C), 122.8, 120.5, 115.3, 114.8, 103.2, 98.2, 93.5, 33.3, 31.7 (2C), 29.7, 29.5, 29.3, 26.9, 24.7, 22.5, 13.5. Anal. Calcd. for C_33_H_42_O_8_: C, 69.94; H, 7.47. Found: C, 69.63; H, 7.44 (Figures S1 and S2).

#### 2.1.4. General Procedure for the Synthesis of Compounds **1**,**4**,**5**,**6**

In a round-bottom flask, to a solution of **8** (to obtain **1**), **3** (to obtain **4**), **7** (to obtain **6**), **9** (to obtain **5**) (1 equiv), and OLAC (1 equiv) in 30 mL of acetone (DMSO in the case of **7**), 260 mg of PPL was added. The temperature was maintained at 37 °C, with an agitation rate of 130 rpm. After 48 h, the mixture was filtered, washed with a cold solution of NaHCO_3_, and extracted with Et_2_O to afford the corresponding oleate.

##### 2-(3,4-dihydroxyphenyl)-5,7-dihydroxy-4-oxo-4*H*-chromen-3-yl-octadec-9-enoate (Compound **1**)

Yellow resin, 88% yield. ^1^H-NMR (300 MHz, DMSO-*d_6_*) δ 7.78 (d, 1H*, J* = 1.9 Hz), 7.64 (dd, 1H dd, *J* = 1.7 Hz, 8.5 Hz), 6.98 (d, 1H*, J* = 8.5 Hz), 6.50 (d, 1H, *J* = 1.3 Hz), 6.28 (d, 1H, *J* = 1.4 Hz), 5.42–5.38 (m, 2H), 2.29 (t, 2H, *J* = 7.3 Hz), 2.05 (m, 4H), 1.57 (m, 2H), 1.34–1.24 (m, 20H), 0.94 (m, 3H). ^13^C NMR (DMSO-*d_6_*) δ 176.2, 174.8, 164.3, 161.1, 156.5, 148.1, 147.2, 145.4, 136.1, 130.0, 129.9, 122.4, 120.3, 115.9, 115.4, 103.4, 98.5, 93.7, 34.0, 31.7, 29.5, 29.4, 29.2, 29.1, 29.0, 28.9, 28.9, 26.9, 24.9, 22.5, 14.4, 14.2 (Figures S3 and S4).

##### 5-hydroxy-4-oxo-2-phenylchroman-7-yl oleate (Compound **4**)

White resin, 84% yield. ^1^H-NMR (CDCl_3_) δ 12.1 (bs, 1H), 7.6–7.3 (m, 5H), 6.0–5.9 (m, 2H), 5.6–5.5 (m, 3H), 3.2–3.1 (m, 2H), 2.8–2.7 (m, 2H), 2.1–1.9 (m, 4H), 1.6–1.4 (m, 2H), 1.4–1.2 (m, 20H), 0.89 (t, 3H, *J*= 3.6 Hz.). ^13^C NMR (CDCl_3_) δ 195.5, 175.9, 163.1 (x2C), 157.0, 129.9 (2C), 129.6 (2C), 128.8 (2C), 126.0, 98.0, 97.0, 96.7, 79.1, 43.2, 31.8, 29.7, 29.6 (2C), 29.4 (3C), 29.3, 29.2, 29.1, 29.0 (2C), 24.8, 22.6, 14.0. Anal. Calcd. for C_33_H_42_O_8_: C, 69.94; H, 7.47. Found: C, 69.72; H, 7.46 (Figures S5 and S6).

##### 2-(3,4-Dihydroxyphenyl)-5,7-dihydroxychroman-3-yl oleate (Compound **5**)

Light orange resin, 65% yield. ^1^H NMR (Acetone-*d_6_*): δ 6.81–6.79 (m, 1H), 6.70–6.60 (m, 2H), 5.95 (d, 1H, *J* = 2.07 Hz), 5.80 (d, 1H, *J* = 2.07 Hz), 5.33–5.20 (m, 4H), 2.90–2.78 (m, 2H), 2.25 (t, 2H, *J* = 7.3 Hz), 2.05–1.80 (m, 4H), 1.55–1.40 (m, 2H), 1.39–1.10 (m, 20H), 0.88 (t, 3H, *J* = 8.0 Hz). ^13^C NMR (Acetone-*d_6_*): 174.0, 156.7, 155.9, 144.7 (2C), 131.5, 129.6, 129.5, 119.1, 114.7, 114.3, 99.6, 95.2, 94.5, 81.7, 67.4, 33.3, 31.7, 29.7 (x 2C), 29.4 (3C), 29.3, 29.2, 29.0, 27.84 (2C), 26.8, 24.7 (2C), 22.4, 13.4. Anal. Calcd. for C_33_H_46_O_7_: C, 71.45; H, 8.36. Found: C, 71.32; H, 8.39 (Figures S7 and S8).

##### 2-(2,4-dihydroxyphenyl)-5,7-dihydroxy-4-oxo-4*H*-chromen-3-yl oleate (Compound **6**)

Phosphorescent yellow resin, 62% yield. ^1^H NMR (DMSO-*d_6_*): δ 12.50 (bs, 1H), 10.88 (bs, 1H), 10.19 (bs, 1H), 9.45 (bs, 1H), 8.05 (d, 1H, *J* = 6.96 Hz, 9.07 Hz), 6.91 (dd, 1H, *J* = 6.96 Hz, 9.07 Hz), 6.55 (d, 1H, *J* = 2.08 Hz), 6.28 (d, 1H, *J* = 2.06 Hz), 5.38–5.25 (m, 2H), 2.18 (t, 2H, *J* = 7.3 Hz), 2.01–1.92 (m, 4H), 1.50–1.40 (m, 2H), 1.38–1.17 (m, 20H), 0.85 (t, 3H, *J* = 8.0 Hz). ^13^C NMR (DMSO-*d_6_*): δ 175.6, 174.3, 167.8, 166.4, 164.3, 161.1, 159.6, 146.1, 144.1, 136.6, 129.9 (x2C), 115.8, 108.8, 108.4, 104.0, 103.0, 98.3, 94.0, 34.1 (x2C), 31.6, 29.5, 29.4, 29.2, 29.0, 28.9 (x 2C), 28.8, 26.97, 24.8, 22.4, 14.3. Anal. Calcd. for C_33_H_42_O_8_: C, 69.94; H, 7.47. Found: C, 69.72; H, 7.46 (Figure S9).

### 2.2. Cell Culture

Human colorectal (HCT 116, ATCC^®^ CCL-247™) and HT-29 (ATCC^®^ HTB-38™) adenocarcinoma cells, human prostate (PC-3, CRL-1435™) and mammary gland (MCF7, HTB-22™) adenocarcinoma cells, human hepatocellular Hep G2 [HEPG2] (ATCC^®^ HB-8065™) carcinoma cells, as well as human mammary metastatic MDA-MB-231 (ATCC^®^ HTB-26™) cells were purchased from *The American Type Culture Collection* (ATCC, Manassas, VA, USA). DLD-1 adenocarcinoma cells were purchased from Horizon Discovery Ltd. The HCT116 p53-/- cell line was purchased from Johns Hopkins University. HCT116, DLD-1, and HT-29 cells were maintained in RPMI (PAN Biotech GmbH) with a 10% fetal bovine serum (PAN Biotech GmbH), PC-3 cells were maintained in RPMI (PAN Biotech GmbH) with a 10% fetal bovine serum (PAN Biotech GmbH, Aidenbach, Germany) and 1% sodium pyruvate (Sigma-Aldrich, Darmstadt, Germany). MCF7 and MDA-MB-231 cells were maintained in DMEM (Gibco) with a 10% fetal bovine serum (PAN Biotech GmbH), 1% non-essential amino acids (Gibco), and 1% sodium pyruvate (Sigma-Aldrich). Hep G2 cells were maintained in DMEM (Gibco) with a 10% fetal bovine serum (PAN Biotech GmbH). The HCT116 p53-/- cell line was maintained in McCoy’s 5A media (Gibco) with a 10% fetal bovine serum (PAN Biotech GmbH). All media were supplemented with penicillin (100 U/mL)/streptomycin (100 mg/mL) (PAN Biotech GmbH). All cell lines were cultured in T75 flasks (TPP) at 37 °C, 5% CO_2_ in a humidified incubator according to the standard procedures. Cells were passaged every 2-3 days and used in experiments when being approximately 80% confluent. Human normal intestinal epithelial cell line HCEC was used to evaluate cytotoxicity against normal tissue and was originally a gift from Dr. Pablo Steinberg (Max-Rubner Institute, Karlsruhe, Germany). The mycoplasma free status was verified regularly. Cell lines were authenticated using the Multiplex Cell Authentication by Multiplexion (Multiplexion, Heidelberg).

### 2.3. Preparation of Cells for Experiments

For cell fitness screening and high-content imaging experiments 15,000 and 5000 cells, respectively, suspended in 45 µL of DMEM, supplemented with a 10% fetal bovine serum and penicillin (100 U/mL)/streptomycin (100 mg/mL) were plated into each well of a 384-well plate (BD Falcon) and were cultured overnight at 37 °C, 5% CO_2_ in a humidified incubator. Dispensing steps were performed manually with a multichannel pipet (Eppendorf). Compounds were added to the cells the next day.

### 2.4. Crystal Violet Assay

To investigate the potential of the compounds to alter cell viability, the crystal violet assay was performed as described previously [30]. A total of 7500 cells were seeded in 100 µL of a cell culture medium per well of a 96-well plate (Corning) and allowed to adhere for 24 h at 37 °C and 5% CO_2_. Afterwards, cells were treated with the respective compound at various concentrations for 48 h. Then, cells were stained with crystal violet for 15 min at rt. Absorbance of stained cells was measured photometrically at 595 nm using a multilabel reader Victor^®^ X3 (Perkin Elmer, Velbert, Germany).

### 2.5. Detection of Apoptosis via the Annexin V Assay

Flow cytometry was used to detect apoptosis using the Annexin V-FITC kit (Miltenyi Biotec) according to the manufacturer’s protocol. In addition, 5.5 × 10^5^ cells were seeded in 6 cm cell culture dishes (Corning) and allowed to adhere for 24 h at 37 °C and 5% CO_2_. Afterwards, cells were treated with 50 µM of the respective compound. After 48 h, floating and adherent cells were harvested by trypsinization. Then, cells were centrifuged, washed with a binding buffer, resuspended in 100 µL of a binding buffer, and stained by adding 10 µL of the Annexin V-FITC solution for 15 min at rt in the dark. Cells were washed again and resuspended in a 500 µL binding buffer. A staining solution of 5 µL of propidium iodide (PI) were added immediately prior to the analysis by flow cytometry in a FACSCanto II (BD Biosciences) and FlowJo software (v10.5.3, FlowJo LCC, Franklin Lakes, NJ, USA).

### 2.6. Western Blotting

The Western Blot analysis was performed as described previously [31]. To determine the alterations of protein levels, 40 µg of total protein were separated by 12% and by 7.5% denaturing SDS-PAGE and transferred onto 0.2 µm nitrocellulose membranes overnight. The membranes were blocked with a 5% milk powder solution for 1 h and incubated with primary anti-PARP-IgG (1:1.000, Cell Signaling, #9532), anti-H2AX-IgG (1:10.000, Millipore, #07-627), anti-γH2AX-IgG1 (1:5.000, Millipore, #05-636), anti-cyclin-B1 (1:5.000, Santa Cruz, #sc-752), or anti-phospho-H3 (1:1.000, Cell Signaling, #9701) at 4 °C overnight. The membranes were then incubated with secondary horseradish-peroxidase-coupled antibodies goat-anti-mouse IgG (H+L) (1:10.000, Thermo Fisher Scientific, #A-11029) or goat-anti-rabbit IgG (H+L) (1:10.000, Thermo Fisher Scientific, #31460) for 1 h at rt. GAPDH served as a control for equal loading of the samples, the blots were incubated with HRP-labeled anti-GAPDH (1:100.000, Abnova, #MAB5476) for 45 min at rt. The blots were developed using the Immobilon Western Chemiluminescent horseradish peroxidase (HRP) substrate kit (Merck Millipore). Images were captured using a GeneGnome (Syngene).

### 2.7. Cell Cycle Analysis

Cell cycle analysis was performed as described previously [32]. To determine the cell cycle distribution, 5.5 × 10^5^ cells were seeded in 6 cm cell culture dishes (Corning) and allowed to adhere for 24 h at 37 °C and 5% CO_2_. Afterwards, cells were treated with 50 µM of the respective compound. After 48 h, floating and adherent cells were harvested by trypsinization. Then, cells were centrifuged, washed in PBS, and fixed in 70% ice cold ethanol overnight at 4 °C. The DNA was stained using a staining solution containing 150 µg/mL PI and 0.5 mg/mL RNase for 30 min at rt in the dark. The DNA content was measured by a FACSCanto II (BD Biosciences) and the cell cycle distribution was determined using the FlowJo software (v7.6.5, FlowJo LCC).

### 2.8. Concentration Response Experimentation

A library of partly in-house synthesized molecules was used for compound screening [33]. The library contained eight chemicals dissolved in 100% DMSO to yield 100 mM stock solutions. Prior to the compound screening, serial dilutions containing 0.03, 0.1, 0.3, 1, 3, 10, 30, 100, 300, and 1000 µM chemicals were prepared from stocks in RPMI Invitrogen, distributed into the wells of 96-well plates (TPP) and stored at 4 °C until the experiment. All dispensing steps were performed using a liquidator liquid handling system (Steinbrenner). The compound treatment was performed by adding 5 µL of the drug solution to each well of a 384-well plate using a liquidator liquid handling system resulting in 0.003, 0.01, 0.03, 0.1, 0.3, 1, 3, 10, 30, and 100 µM final concentration. Plates were incubated for 48 h at 37 °C, 5% CO_2_, and subsequently used in cell fitness screening.

### 2.9. Cell Fitness Indicator

*CellTiter-Glo Luminescent Cell Viability Assay Kit* (Promega). The technique is used to assess the number of metabolically active cells in a culture based on the measurement of intracellular ATP. The method involves the addition of a single reagent to the cells resulting in cell lysis and emission of luminescence light, which is proportional to the amount of intracellular ATP. The luminescence signal is considered to be directly proportional to the total number of cells in the culture.

### 2.10. Cell Fitness Screening

Prior to evaluating the cell fitness using the *CellTiter-Glo Luminescent Cell Viability Assay Kit* the staining solution was entirely removed from the wells by turning the plate upside-down onto a stack on tissue paper. The *CellTiter-Glo Luminescent Cell Viability Assay Kit* was employed according to the manufacturer’s instructions and was diluted in DMEM without phenol-red (Invitrogen) in a 1:1-ratio. In addition, 10 µL of the mixture was pipetted to the cells and was incubated for 10 min in the dark at an ambient temperature. Subsequently, the intensity of the luminescence signal was quantified using a VICTOR™ X4 plate reader (Perkin Elmer, Velbert, Germany) (exposure time: 0.1 s, no filter). All pipetting steps were conducted with a Multidrop Combi dispensing system (Thermo, Waltham, MA, USA). Screens were performed in quadruplicate.

### 2.11. High-Content Imaging

Compounds **1**, **2**, **4**, **5**, and **7**, which showed dose-dependent effects in concentration-response viability screening experiments were re-screened 100 µM in HCT 116 cells. DMSO and 100 µM of 5-fluorouracil were used as a negative and positive control, respectively. Compound exposure was terminated by paraformaldehyde fixation and subsequent immunostaining using α-tubulin (FITC-labeled, diluted 1:500, F2168, Sigma-Aldrich, Darmstadt, Germany) and α-actin (TRITC-phalloidin, diluted 1:3000, P1951, Sigma-Aldrich, Darmstadt, Germany) antibodies, as well as Hoechst 33342 DNA stain (diluted 1:8000, Sigma-Aldrich, Darmstadt, Germany). All pipetting steps were conducted with a Multidrop Combi dispensing system (Thermo). Cells were imaged with a Nikon Eclipse Ti imaging system (Nikon, Japan) using a 20× objective (CFI Plan Fluor DL 20X Phase, Nikon, Japan). Excitation light from a xenon lamp (Lambda LS, Sutter Instruments, Novato, CA, USA), filtered through a filter block (C-FL Epi-FL FITC, EX 465-495, DM 505, BA 515-555, Olympus, Japan) was used to excite and measure the fluorescence signal. Fluorescence was imaged by a sCMOS camera (NEO, Andor, Ireland) and digitized to a disk onto a personal computer with a Windows 10 Operating System (Microsoft Corporation, Redmond, WA, USA). The original resolution of the camera was 2560 × 2160 pixels, however, images were binned (2 × 2), resulting in a final resolution of 1280 × 1080 pixels.

### 2.12. Image Analysis and Quantification of Cell Number

Fluo-micrographs of cells were segmented and quantitatively analyzed using an improved version of DetecTIFF^®^ suite. In brief, images of Hoechst 33342-stained cells were first segmented using an iterative size- and intensity-based [34] thresholding algorithm, and subsequently, the total number of cells was calculated for each image.

### 2.13. Data Analysis

The plate reader data from dose-response experiments with the custom assembled library were annotated in Microsoft Excel and statistically analyzed using Origin (OriginLab). Quantification data was first normalized to the plate median and replicates were subsequently averaged. Concentration-response relationships were fitted using the fit sigmoidal function in Origin. Imaging data was quality controlled by visual inspection to exclude images of cells, which were defocused and cell numbers were averaged from 6-8 images for control and test experiments.

## 3. Results and Discussion

### 3.1. Synthesis

The oleoyl derivatives were synthesized through a bio-catalytic approach, involving PPL as a catalyst. By using PPL, QUE-3-oleate (**1**) was previously obtained with a very high yield, as a unique product, and recycling the enzyme for five times [35]. In this study, the regioselectivity of PPL towards the C-3 OH was confirmed through the reaction of other polyphenols in the same conditions. As reported, this reaction process proceeded with the formation of only one product in high yield. Moreover, PPL is a very inexpensive enzyme, still scarcely used in chemistry. Furthermore, the recycle of the enzyme made the reaction very useful for industrial applications. First, the reaction was performed with Novozyme 435^®^, and even after 120 h the formation of the oleoyl derivative was not detected, according to the study of Kumar et al. [36], who demonstrated how Novozyme 435^®^ is unable to acylate polyphenol aglycon forms. The reaction was also conducted in the presence of Lipase B *Candida antarctica*, a recombinant from *Aspergillus oryzae* producing **1** with a low yield (43%). To validate the proposed reaction conditions [35], other polyphenols were used. In the case of (+)-catechin (**9**), which has a primary alcoholic group, only the C-3 ester (**5**) was obtained, though with a low yield compared to **1**.

**Scheme 1:**
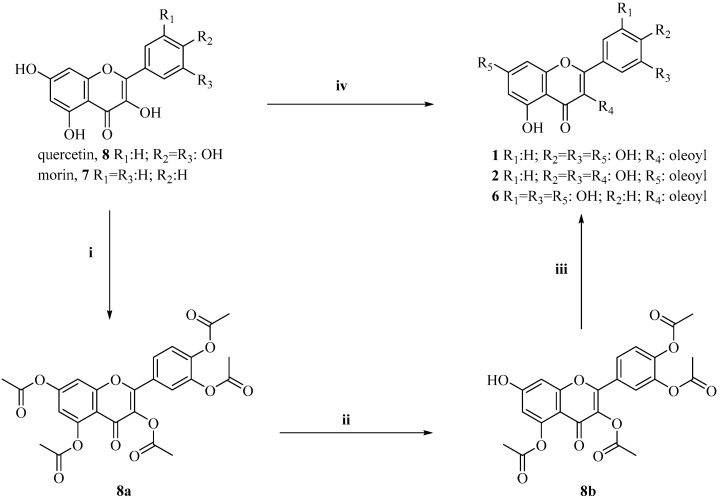
Preparation of oleoyl hybrids of morin and QUE. (i) Acetic anhydride, pyridine, reflux, 5 h; (ii) imidazole, DCM, −15 °C, then rt, 2 h; (iii) *i*) OLAC, PPL, 37 °C, 48 h, *ii*) aqueous HCl 6N, CH_3_CN, reflux, 1.5 h; (iv) OLAC, PPL, 37 °C, 48 h.

**Scheme 2:**
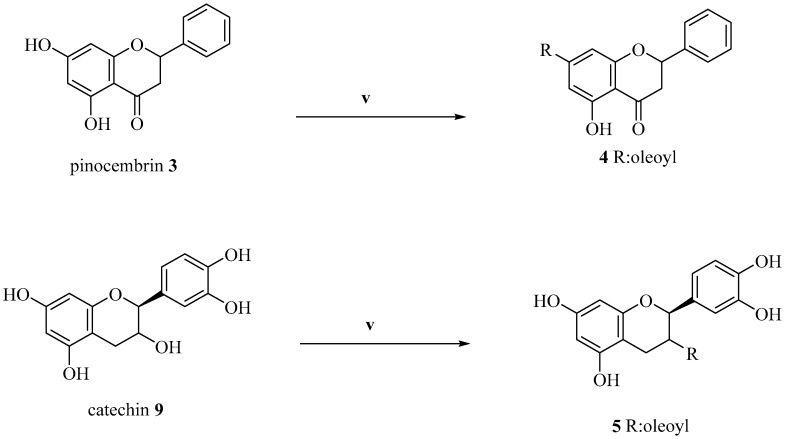
Preparation of oleoyl hybrids of pinocembrin and catechin. v) OLAC, PPL, 37 °C, acetone (DMSO for **7**), 48 h.

In this field, the reaction was also conducted with morin (**7**), a molecule without the catechol group and presence of the C-3 hydroxyl group. In this case, the reaction was very difficult because of its poor solubility in acetone, THF, and *t*-BuOH. The reactions proceeded slowly in all the cases and with very poor yields (<20%). The best solvent for the reaction resulted in DMSO; the yield of compound **6** in this case was 65%. In all the cases, only the C-3 ester was obtained (Scheme 1). Finally, when pinocembrin (**3**) was used, the formation of the ester occured obviously, in a C-7 position, with a moderate yield. These considerations demonstrated how PPL is a good catalyst for the formation of polyphenols-acyl hybrids, although new reactions, with various polyphenols or fatty acids and varying conditions are necessary to fully understand the selectivity and usefulness of the enzyme (Scheme 2). In the case of compound **2**, the C-7 oleoyl derivative of QUE, another synthetic strategy was used; in particular, all the hydroxyl groups of QUE were acetylated (**8a**). After that, in the presence of imidazole, there was the selective cleavage of the acetyl group in a C-7 position, furnishing **8b**, that was subjected to PPL acylation to obtain the oleoyl derivative as a crude intermediate, which furnished the pure compound **2** in acidic conditions (Scheme 1).

### 3.2. Biological Evaluation

**Figure 2:**
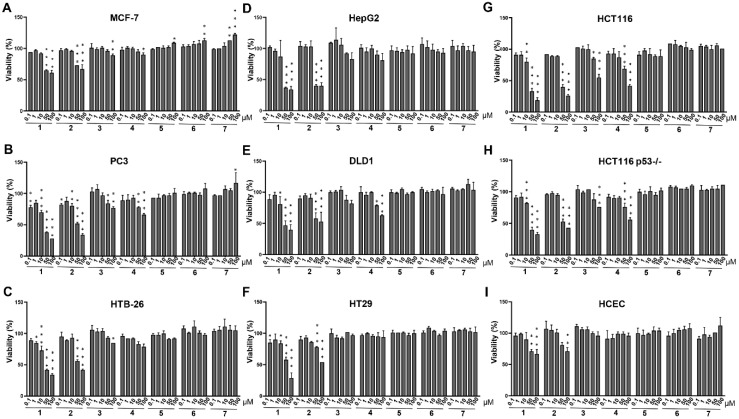
Cell viability analysis using the crystal violet assay. Viability of a panel of eight cancer cell lines (**A**–**H**) and a normal intestinal epithelial cell line (HCEC) (**I**) after treatment with compounds **1**–**7** at different concentrations (0.1–100 µM) for 48 h. Cell viability was shown as the percentage of DMSO control (mean ± SEM). Significant differences between treatment doses and IC_50_ values were calculated by one way ANOVA (GraphPad); * *p* < 0.05, ** *p* < 0.01, *** *p* < 0.001. Values are means of two biological duplicates measured in three technical replicates each (*n* = 6).

**Figure 3:**
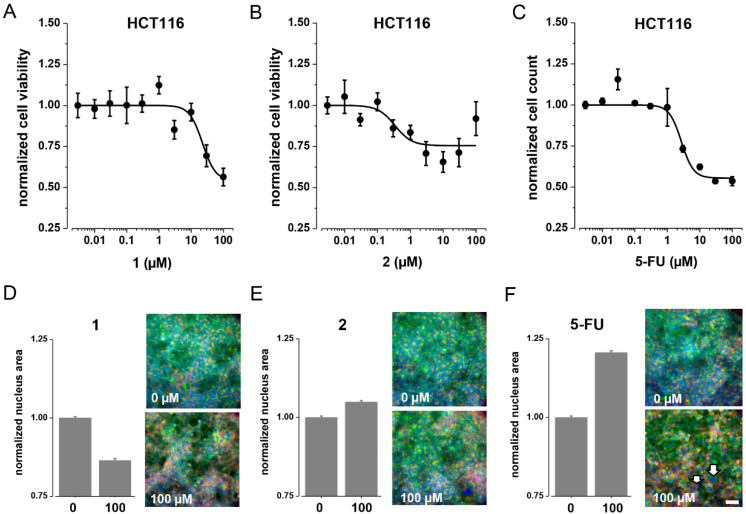
ATP-based and high-content imaging-based viability analysis. (**A**,**B**) Averaged and normalized dose-responses (mean ± SEM, *n* = 8) of compounds **1** and **2** in HCT116 cells, analyzed using the CellTiter Glo^©^ Cell Viability Assay upon 48 h of drug exposure. (**C**) Averaged and normalized dose-response (mean ± SEM, *n* = 8) of 5-FU in HCT116 cells, analyzed based on the cell number derived from Hoechst 33342-labelled cells visualized by high-content fluorescence microscopy. Black lines in **A**–**C** indicate sigmoidal fits applied to calculating the IC_50_ for the tested molecules. (**D**–**F**) Analysis of the nucleus size of HCT116 cells, exposed to compounds **1**, **2**, and 5-FU, respectively. Cells were treated with test molecules for 48 h, fixed, stained with fluorescence indicators to visualize DNA (Hoechst 33342, blue), β-Tubulin (FITC-conjugated anti- β-Tubulin antibody, green), and actin (Rhodamine-Phalloidin, red), and were subsequently visualized using a 20× objective of a high-content microscope. The histograms show the relative nucleus area (mean ± SEM, *n* = 8530–12550, calculated from eight images each), for cells exposed to a 100 µM compound concentration normalized to the control (0 µM, shuttle control). The images represent exemplary fluo-micrographs used for the calculation of the nucleus area and were merged from the aforementioned three fluorescence channels. The arrows in the image in **G** highlight cells with increased nuclei as compared to the control. Scale bar: 100 µm for all immunofluorescence images.

To evaluate the cytotoxicity of the seven hybrid compounds against a panel of cancer cell lines, the crystal violet assay was used (Figure 2). The IC_50_ values are given in Table 1. The most potent anticancer effects were recorded for compounds **1** and **2** that from a chemical structural point of view, are two regioisomers, differing only by the position of one OH group on ring B. Both compounds revealed IC_50_ values between 10 and 50 µM in all cell lines. For these reasons, compounds **1** and **2** were subjected to a more detailed functional analysis. Interestingly, MCF7 cells showed the lowest sensitivity. This cell line is well known as a low aggressive breast cancer cell line suggesting that proliferation might be necessary for the mechanism of action. Both compounds were very effective against the highly aggressive breast cancer cell line HTB-26, against the pancreatic cancer cell line PC-3, and the hepatocellular carcinoma cell line HepG2 with IC_50_ values between 10 and 50 µM (Table 1). Both compounds were obviously less active towards normal intestinal epithelial cell line HCEC suggesting tumour selective treatment effects (Figure 2I). To better address the utility of these hybrids, that comprise a backbone typical of those present in the foods, in the cancer management, we further focused on their anti-proliferative activity against colorectal cancer cells. When considering the panel of colorectal cancer cell lines, no p53 dependent anticancer effects seemed to exist since tumour cells with a p53 mutation such as DLD1 and HT29 showed the same response as tumour cells with a complete loss of p53 or HCT116 p53 wildtype cells (Figure 2E–H). Given the fact that more than 50% of colon carcinoma show a mutant p53 status, the two hybrids might be very promising to overcome resistance in tumours with a functional p53 loss. Exemplarily, we used HCT116 cells and tested both compounds and the clinically used drug 5-FU in an assay based on Hoechst 33342-labelled cells visualized by high-content fluorescence microscopy (Figure 3A–C).

Interestingly, we could show that both compounds, preferentially compound **2**, have comparable cytotoxic effects as 5-FU in HCT116 cells: The IC_50_ value for compound **1** was 22.4 µM and for compound **2** it was 0.34 µM. The IC_50_ value for 5-FU was 2.7 µM (Figure 3A–C). When analyzing the size of the cell nuclei using triple immunofluorescence staining, we could confirm the well-known S-phase arrest in 5-FU treated cells reflected by a significantly increased size of cell nuclei (Figure 3D–F). Compound **1** induced a significant decrease in nuclei size suggesting chromatin condensation as a sign for apoptosis induction, whereas compound **2** did not change the size of nuclei (Figure 3D,E).

**Figure 4:**
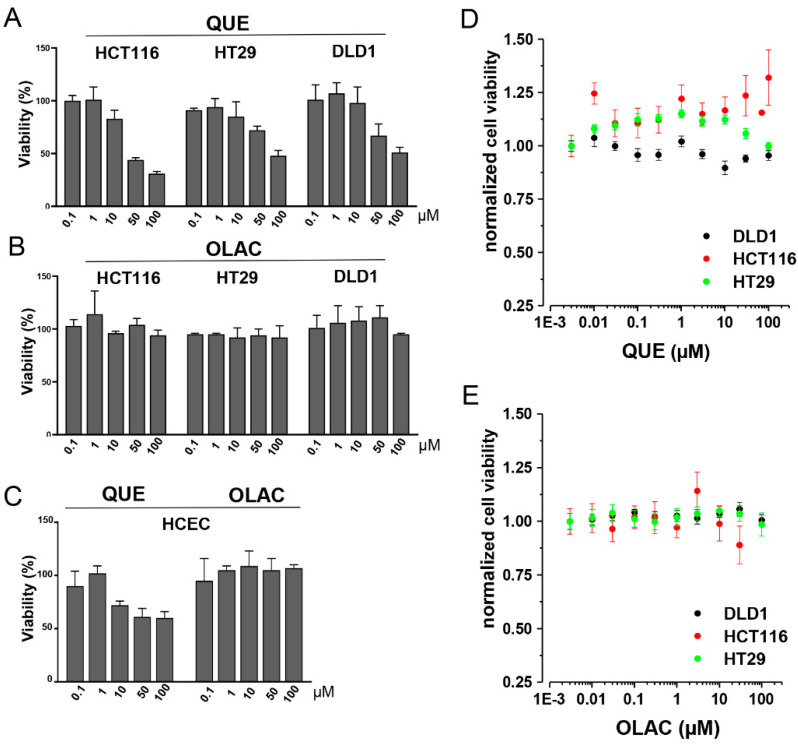
Viability effects of QUE and OLAC. (**A**–**C**) Viability analysis using the crystal violet assay in three colon cancer cell lines (**B**,**C**) and in a normal intestinal epithelial cell line (HCEC). (**C**) After the treatment with QUE and OLAC at different concentrations (0.1–100 µM) for 48 h. Cell viability was shown as a percentage of DMSO control (mean ± SEM). Significant differences between the treatment doses and IC_50_ values were calculated by one way ANOVA (GraphPad); * *p* < 0.05, ** *p* < 0.01, *** *p* < 0.001. Values are means of two biological duplicates measured in three technical replicates each (*n* = 6). (**D**,**E**) ATP-based cell viability analysis: Dose-responses (mean ± SEM, *n* = 8) of the compounds QUE and OLAC in DLD1 (black), HCT116 (red), and HT29 cells (green), respectively. The anti-proliferative activity of the two drugs was evaluated using the CellTiter Glo^©^ Cell Viability Assay upon 48 h of drug exposure.

Regarding the parental compound QUE, we observed that it was reducing the viability of the tumour cells at doses of 50 and 100 µM but to a lesser extent than compounds **1** and **2** (Figure 2E–G and Figure 4A, Table 1). The pro-apoptotic effects of this flavonoid in colorectal cancer have been already shown [37,38]. However, QUE’s moderate cytotoxicity against normal intestinal epithelial cells presents a potential advantage of our novel compounds **1** and **2** (Figure 4C, Table 1). In contrast, OLAC did not show any cytotoxic effects neither in colon cancer cells nor in normal HCEC cells in our treatment protocol (Figure 4B,C, Table 1). The same results were seen when testing QUE and OLAC in an ATP-based cell viability assay. Both drugs did not show remarkable effects on intracellular ATP concentration (Figure 4D,E). It is worth mentioning that the crystal violet assay quantifies the maintained adherent cells that did not undergo cell death suggesting pro-apoptotic effects when binding of the crystal violet dye decreased after the drug treatment. Nevertheless, the measurement might be potentially compromised by proliferative responses that mutually occur with cell death.

**Figure 5:**
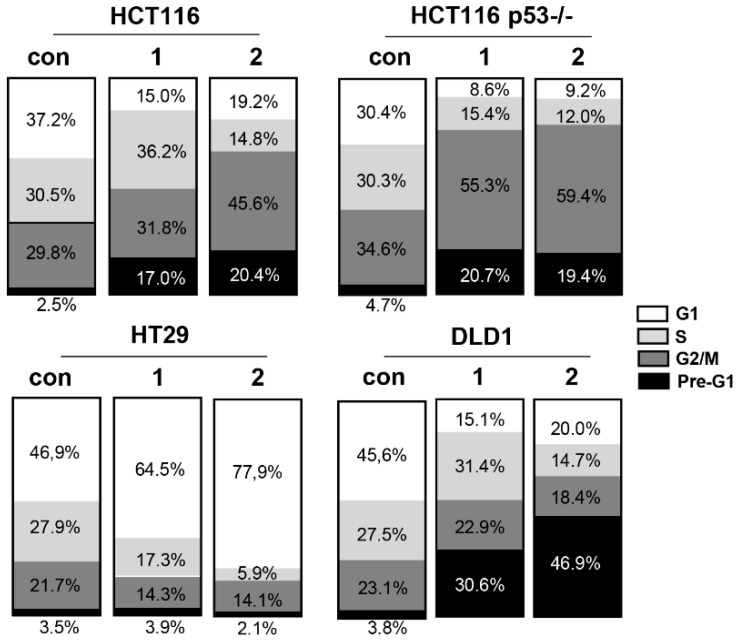
Cell cycle and cell death analysis in four colon cancer cell lines having a different p53 mutation status. Cell cycle analysis using quantification of propidium-iodide staining by flow cytometry after a 48 h treatment with compounds **1** and **2**. The graph shows the percentage of cells in different cell cycle phases: G0/G1 (diploid fraction), S, G2/M (tetraploid fraction), and sub-G1 population representing the apoptotic cell fraction.

**Figure 6:**
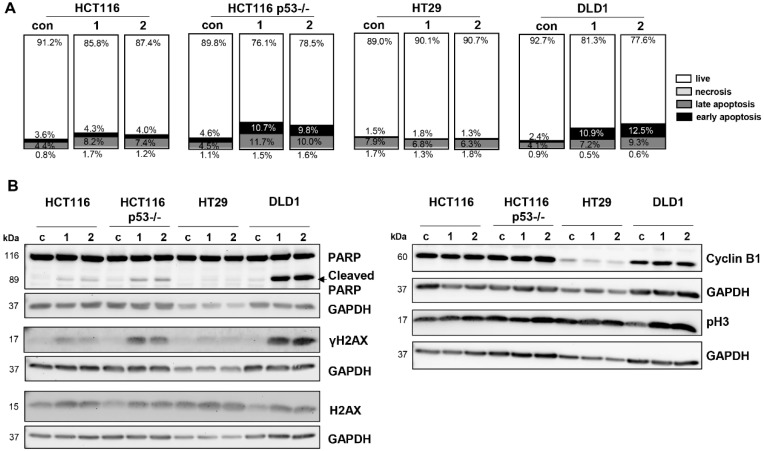
Examination of apoptosis induction in four colon cancer cell lines having a different p53 mutation status. (**A**) Annexin-PI-analysis (biological duplicate, *n* = 2) under a 48 h drug exposure (50 µM) measured by flow cytometry. (**B**) Western Blot analysis (*n* = 2, representative membrane is shown) for the detection of PARP cleavage, ɣH2AX, H2AX, Cyclin B1, and pH3 levels after a 48 h of treatment with 50 µM of compounds **1** and **2**. GAPDH was used as a loading/house keeping control. The size of proteins is given in kDa.

Therefore, in a next step, we examined the effects of compounds **1** and **2** on cell cycle distribution. Indeed, cell cycle analysis showed great differences between the cell lines. There was a remarkable accumulation of G0/G1 phase cells for HT29 cells (Figure 5). This G1 arrest might explain the somewhat conflicting good response in the crystal violet assay where we simply measured crystal violet staining as an equivalent of living cells. The low apoptotic response towards compounds **1** and **2** in HT29 cells was also visible in the Annexin-PI analysis (Figure 6A) and was further verified by a lack of cleaved PARP signals in Western Blotting (Figure 6B).

PARP is a repair protein that detects and repairs DNA damage by binding to DNA breaks. PARP cleavage by caspases destroys its proper repair function and is a hallmark of apoptosis [39]. In a next step, we examined the molecular mechanism of cell death by the Western Blot analysis of the DNA damage marker γ-H2AX, which is massively accumulated when double-stranded breaks occur in the DNA [40]. As expected from the low PARP cleavage, HT29 cells showed the highest resistance against DNA damage induction (Figure 6B). Therefore, we conclude from our data that the treatment with compounds **1** and **2** did not induce cell death in HT29 cells but led to a remarkable stop in proliferation reflecting the well-known resistance of HT29 cells to a plethora of anti-cancer drugs [41,42]. In contrast to the HT29 cells, the HCT116 p53 wildtype and p53-/- cells showed great differences in the distribution of cell cycle populations: When p53 is lost the cells stopped in the G2/M phase after treatment with both compounds, whereas this G2/M arrest was more pronounced with compound **2** (Figure 5).

We suggest that apoptotic cells seemed to exit the cell cycle in the G2 phase since Cyclin B1 as a marker for mitotic entry was not upregulated upon treatment (Figure 6B). Under p53 mutant conditions as given for DLD1 cells, cells seemed to leave the cell cycle in G2/M, since pHH3 as a marker that stains condensed chromatin just before chromosomal segregation was increased for both compounds (Figure 6B). Possibly, the induction of replication errors after treatment with compounds **1** and **2** caused by the lack of the p53 wildtype repair function let the damaged cells leave the cell cycle at G2/M checkpoint. In general, the increase of pre-G1 subpopulation (Figure 5) was well-correlated with an increase in the early and late apoptotic cell population in the Annexin-PI analysis (Figure 6A) and an increase in PARP cleavage (Figure 6B). We suggest that the DNA damage shown as an increase in ɣH2AX signals seems to be the major cause of apoptotic cell death. Indeed, the HCT116 p53-/- cells and p53 mutant DLD1 cells showed the highest apoptotic pre-G1 fraction (Figure 6A) accompanied by the highest ɣH2AX levels (Figure 6B). Interesting experimental data exist, showing that drug-induced p53-dependent downregulation of ɣH2AX leads to the selective survival of normal cells, whereas cancer cells with non-functional p53 seem to be more susceptible to drug-induced damage [43]. Therefore, the induction of massive DNA damage is a critical factor for strong anti-cancer effects. Noteworthy, both compounds did not result in any necrosis induction, which is promising regarding their side effect spectrum.

## 4. Conclusions

The employment of polyphenols as a starting material to develop new treatment tools for colorectal cancer management was examined by a bio-catalytic synthesis of oleoyl-hybrid of QUE, morin, pinocembrin, and (+)-catechin. We demonstrated convincing data about two novel QUE regioisomer oleoyl-hybrids **1** (C-3 oleoyl) and **2** (C-7 oleoyl) that displayed a highly selective cytotoxicity towards cancer cell lines, being less effective in normal intestinal cells and being more efficient in killing than their reference/parental compounds. We observed diverse effects on cell cycle distribution, which seemed to be at least partially dependent on the p53 mutation status of tumour cells. As the mechanism of action for apoptosis induction, we confirmed a massive induction of DNA damage. We suggest the power of these secondary metabolites, appreciated for their antioxidant properties and normally introduced by diet, together with OLAC, to fight human cancers, suggesting also a preventive role.

## Figures and Tables

**Table 1:** IC_50_ values for compounds **1** and **2** in various cancer cell lines and a normal intestinal epithelial cell line.

Cell Lines	Compounds—IC_50_ Values in µM				
	1	2	4	QUE	OLAC
MCF7	>100	>100	>100	na	na
PC3	23.2	51.4	>100	na	na
HTB26	33.8	69.1	>100	na	na
HepG2	41.3	53.2	>100	na	na
DLD1	51.4	>100	>100	98.2	>100
HT29	53.9	>100	>100	>100	>100
HCT116	22.4	0.34	85.3	43.5	>100
HCT116 p53-/-	38.9	68.5	>100	na	na
HCEC	>100	>100	>100	>100	>100

Na: Not analyzed.

## Supplementary Materials

The following are available online at https://www.mdpi.com/2076-3921/9/11/1077/s1, Figure S1: ^1^H NMR of compound **2**, Figure S2: ^13^C NMR of compound **2**, Figure S3: ^1^H NMR of compound **1**, Figure S4: ^13^C NMR of compound **1**, Figure S5: ^1^H NMR of compound **4**, Figure S6: ^13^C NMR of compound **4**, Figure S7: ^1^H NMR of compound **5**, Figure S8: ^13^C NMR of compound **5**, Figure S9: ^1^H NMR of compound **6**.
