# Supplementary material for: New Oleoyl Hybrids of Natural Antioxidants: Synthesis and In Vitro Evaluation as Inducers of Apoptosis in Colorectal Cancer Cells
Source: Antioxidants (Basel). 2020 Nov 3;9(11):1077. doi: 10.3390/antiox9111077 (PMC7692320; doi:10.3390/antiox9111077)

antioxidants

Supplementary Material

New Oleoyl Hybrids of Natural Antioxidants: Synthesis and in vitro Evaluation as Inducers of Apoptosis in Colorectal Cancer Cells

Gabriele Carullo [1,2,†], Sarah Mazzotta [2,3,4,†], Adrian Koch [5,6], Kristin M. Hartmann [7,8], Oliver Friedrich [7], Daniel F. Gilbert [7], Margarita Vega-Holm [4], Regine Schneider-Stock [5,6,*,‡] and Francesca Aiello [2,*,‡]

1 Department of Biotechnology, Chemistry and Pharmacy, Department of Excellence 2018-2022, University of Siena, Via Aldo Moro 2, 53100 Siena, Italy; gabriele.carullo@unisi.it
2 Department of Pharmacy, Health and Nutritional Sciences, Department of Excellence 2018-2022, University of Calabria, Edificio Polifunzionale, 87036 Rende (CS), Italy
3 Department of Pharmaceutical Sciences, University of Milan Via Luigi Mangiagalli 25, 20133 Milano, Italy; sarah.mazzotta@unimi.it
4 Department of Organic and Medicinal Chemistry, Faculty of Pharmacy, University of Seville, Profesor García González 2, 41071 Seville, Spain; mvegaholm@us.es
5 Instituite of Pathology, University Hospital, Friedrich-Alexander University Erlangen-Nürnberg Universitätsstr. 22, 91054 Erlangen, Germany; adrian.koch@uk-erlangen.de
6 Experimental Tumorpathology, University Hospital, Friedrich-Alexander University Erlangen-Nürnberg Universitätsstr. 22, 91054 Erlangen, Germany
7 Institute of Medical Biotechnology Friedrich-Alexander-University Erlangen-Nürnberg, Paul-Gordan-Str. 3, 91052 Erlangen, Germany; kristin.m.hartmann@fau.de (K.M.H.); oliver.friedrich@fau.de (O.F.); daniel.gilbert@fau.de (D.F.G.)
8 Erlangen Graduate School in Advanced Optical Technologies (SAOT), Friedrich-Alexander-University Erlangen-Nürnberg, Paul-Gordan-Str. 6, 91052 Erlangen, Germany
* Correspondence: regine.schneider-stock@uk-erlangen.de (R.S.-S.); francesca.aiello@unical.it (F.A.)
† G.C. and S.M. serve as co-first authors.
‡ R.S.-S. and F.A. serve as corresponding and senior authors.

Contents

Figure S1. ^{1}H NMR of compound **2**
Figure S2. ^{13}C NMR of compound **2**
Figure S3. ^{1}H NMR of compound **1**
Figure S4. ^{13}C NMR of compound **1**
Figure S5. ^{1}H NMR of compound **4**
Figure S6. ^{13}C NMR of compound **4**
Figure S7. ^{1}H NMR of compound **5**
Figure S8. ^{13}C NMR of compound **5**
Figure S9. ^{1}H NMR of compound **6**

Figure S1. ^{1}H NMR of compound **2**

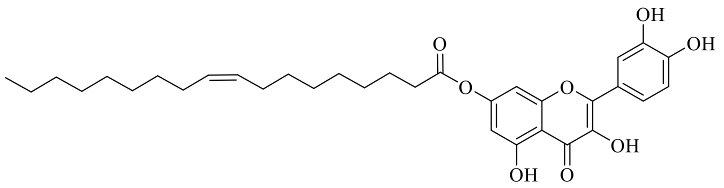

2

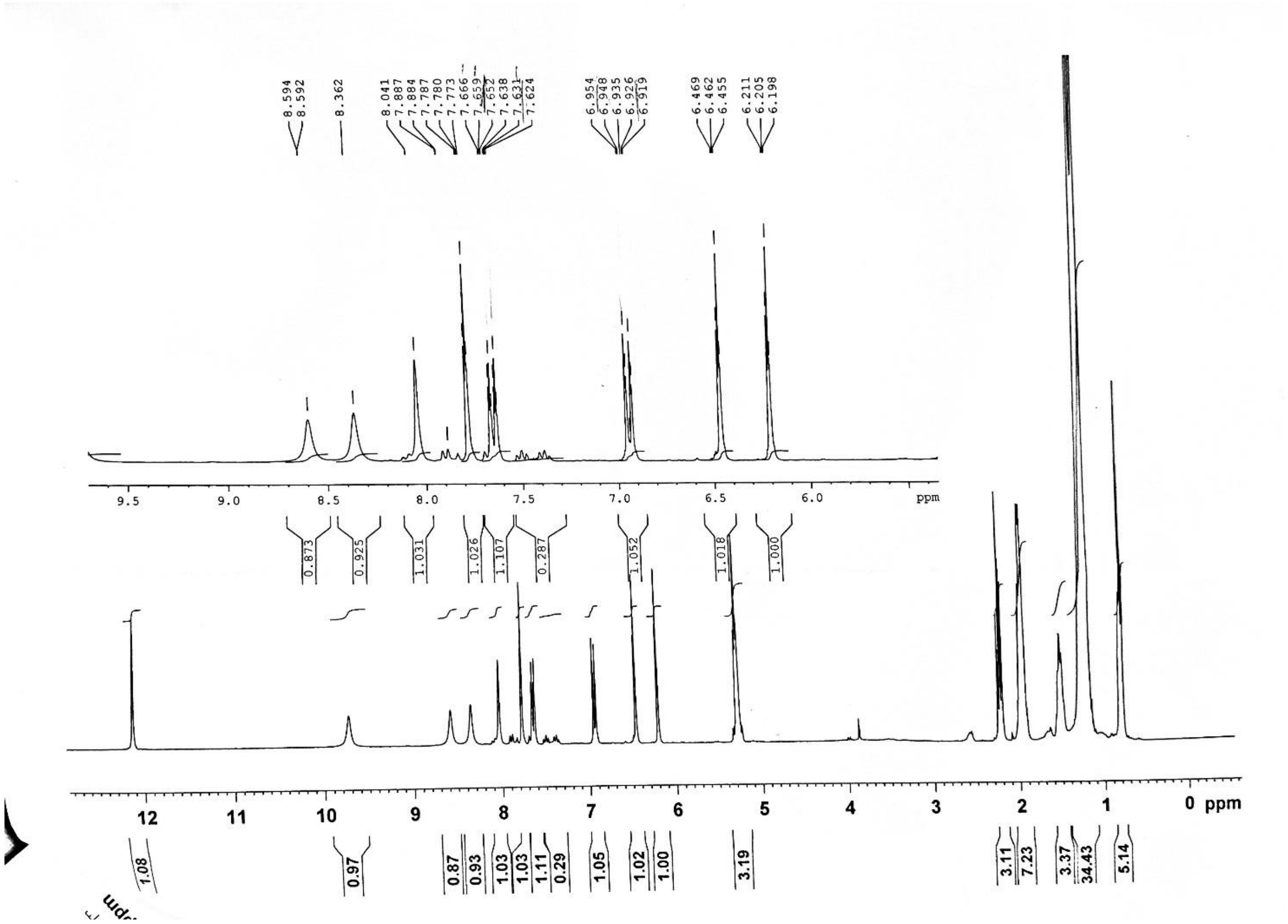

Figure S2. ^{13}C NMR of compound **2**

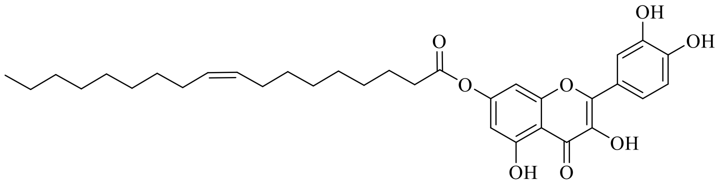

2

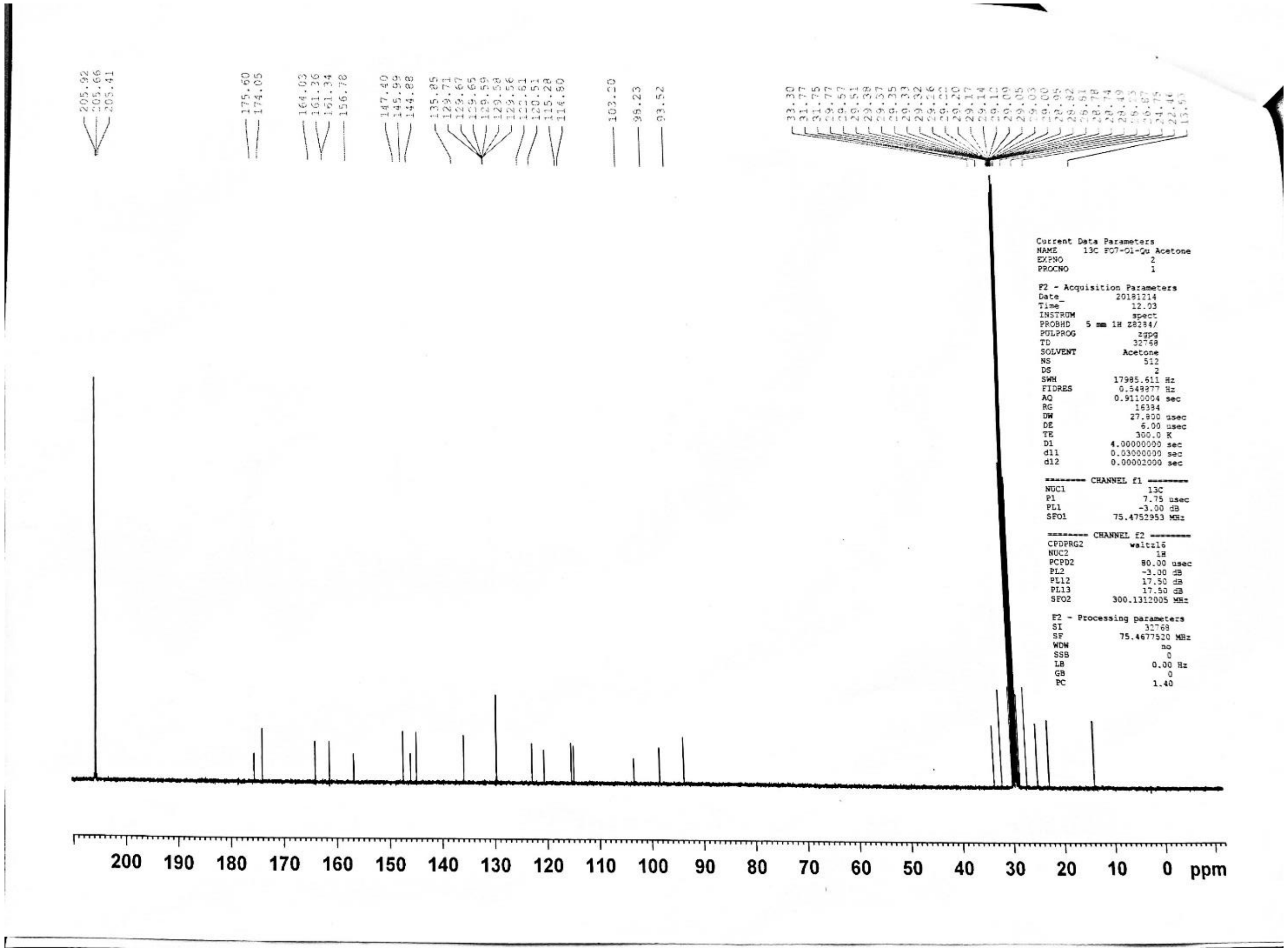

Figure S3. ^{1}H NMR of compound **1**

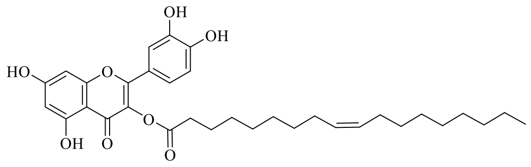

1

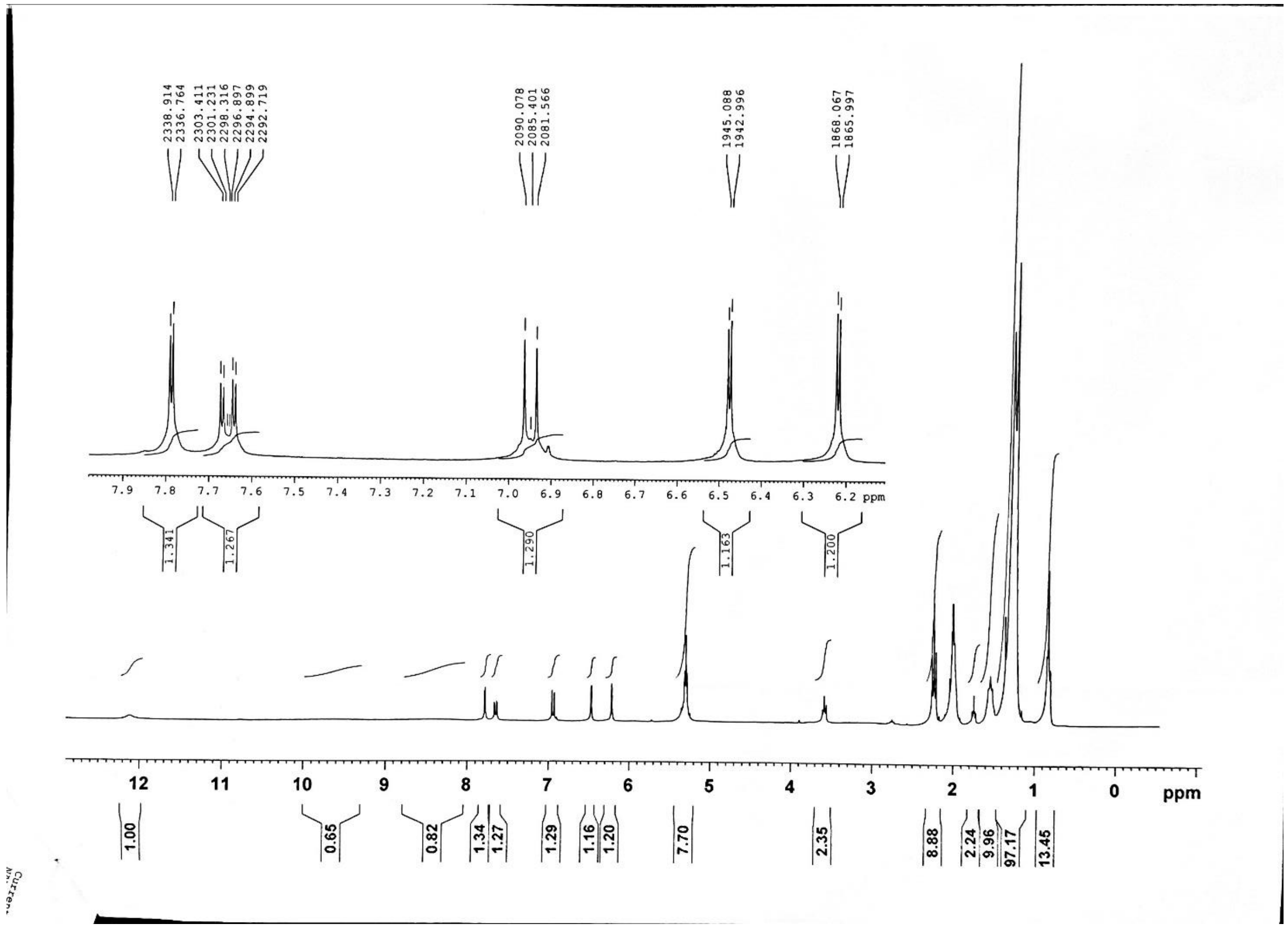

Figure S4. ^{13}C NMR of compound **1**

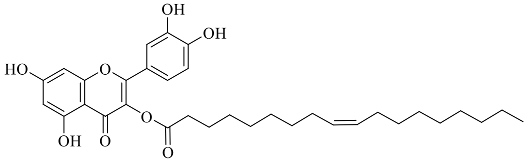

1

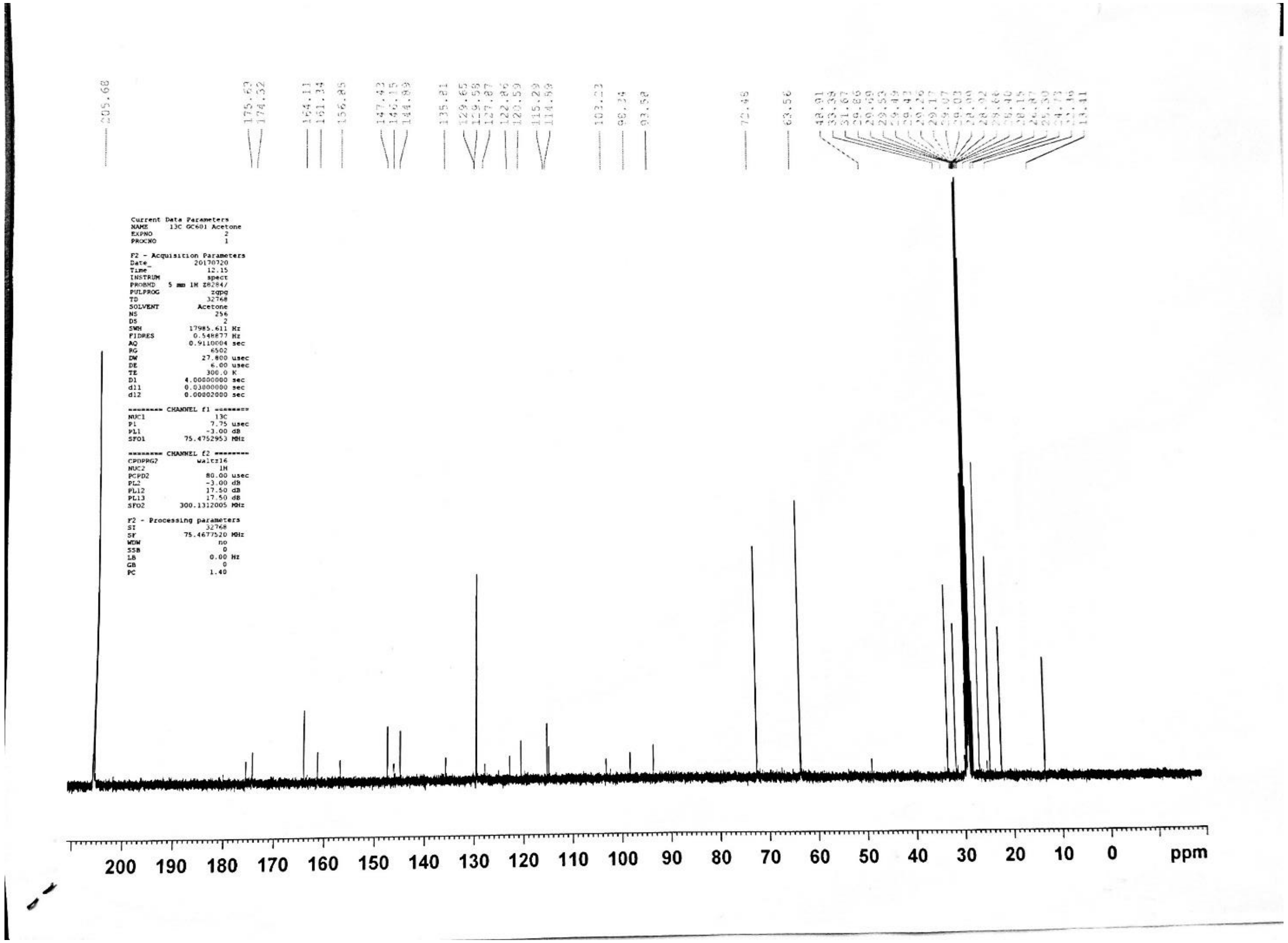

Figure S5. ^{1}H NMR of compound **4**

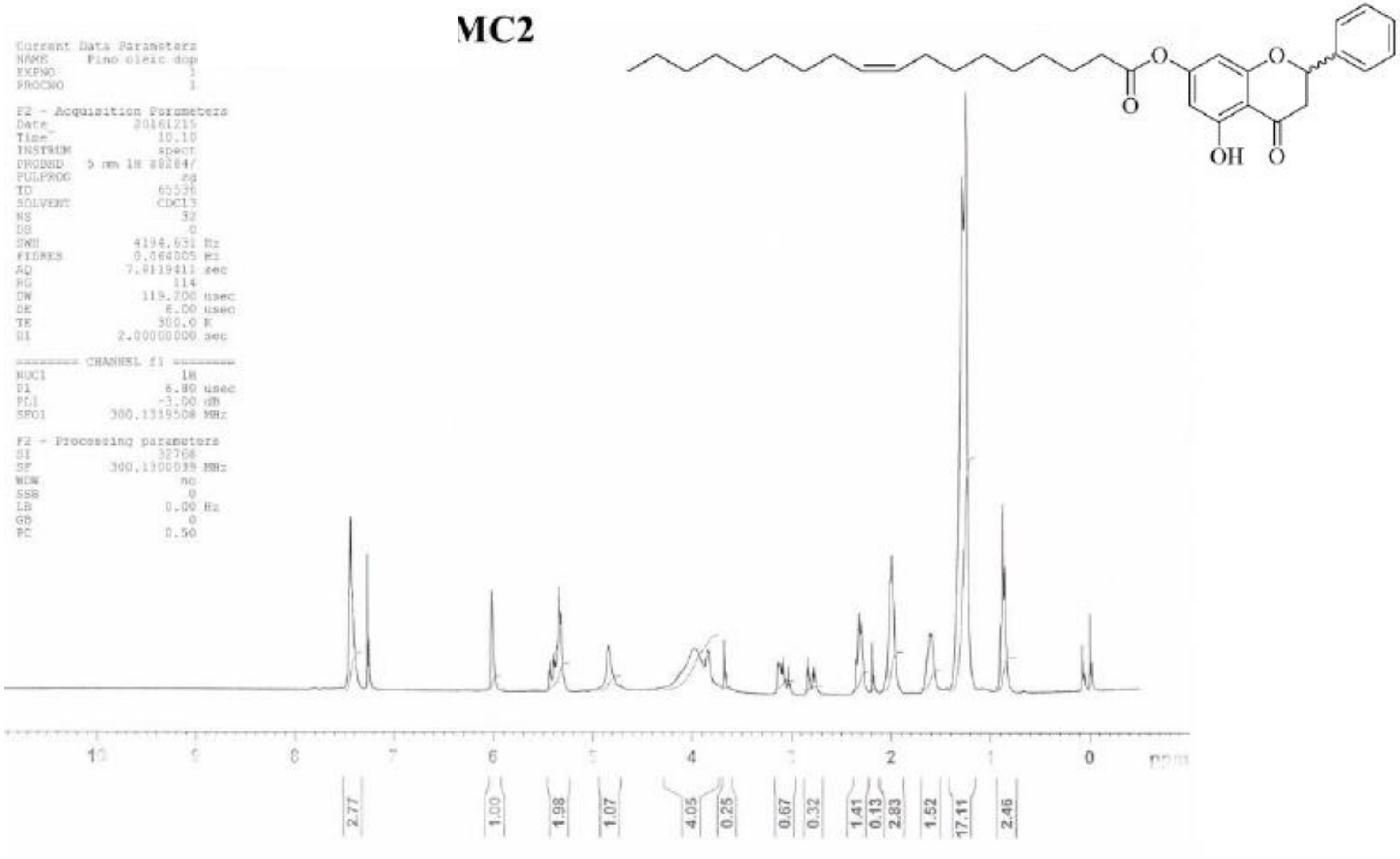

Figure S6. ^{13}C NMR of compound **4**

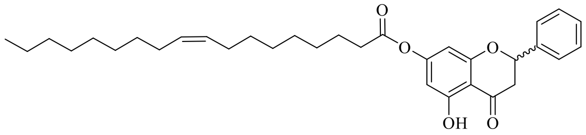

4

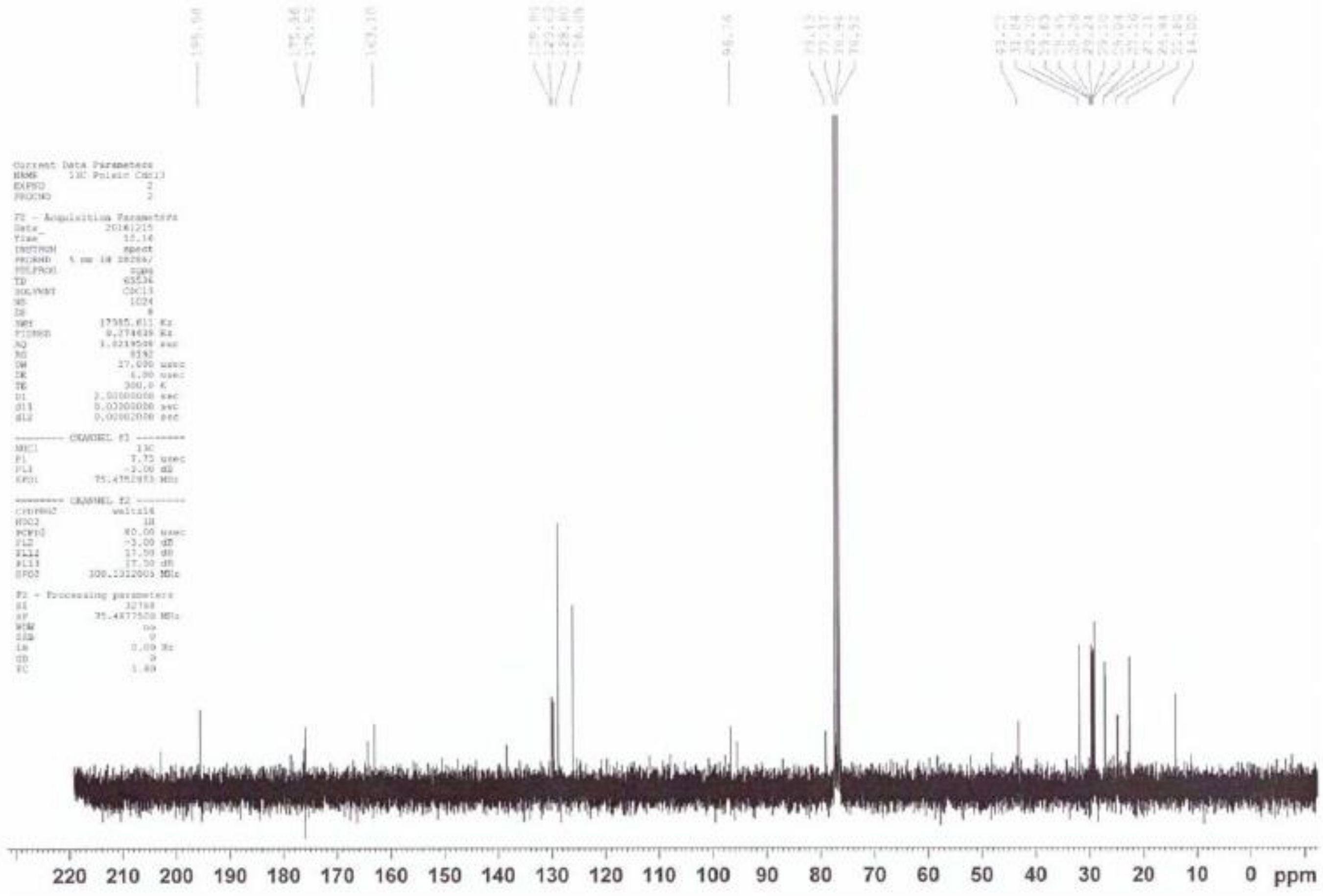

Figure S7. ^{1}H NMR of compound **5**

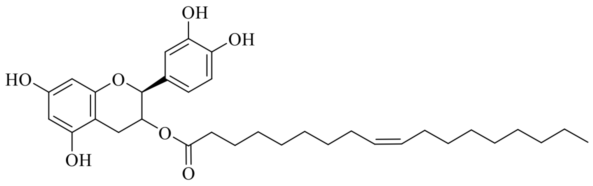

5

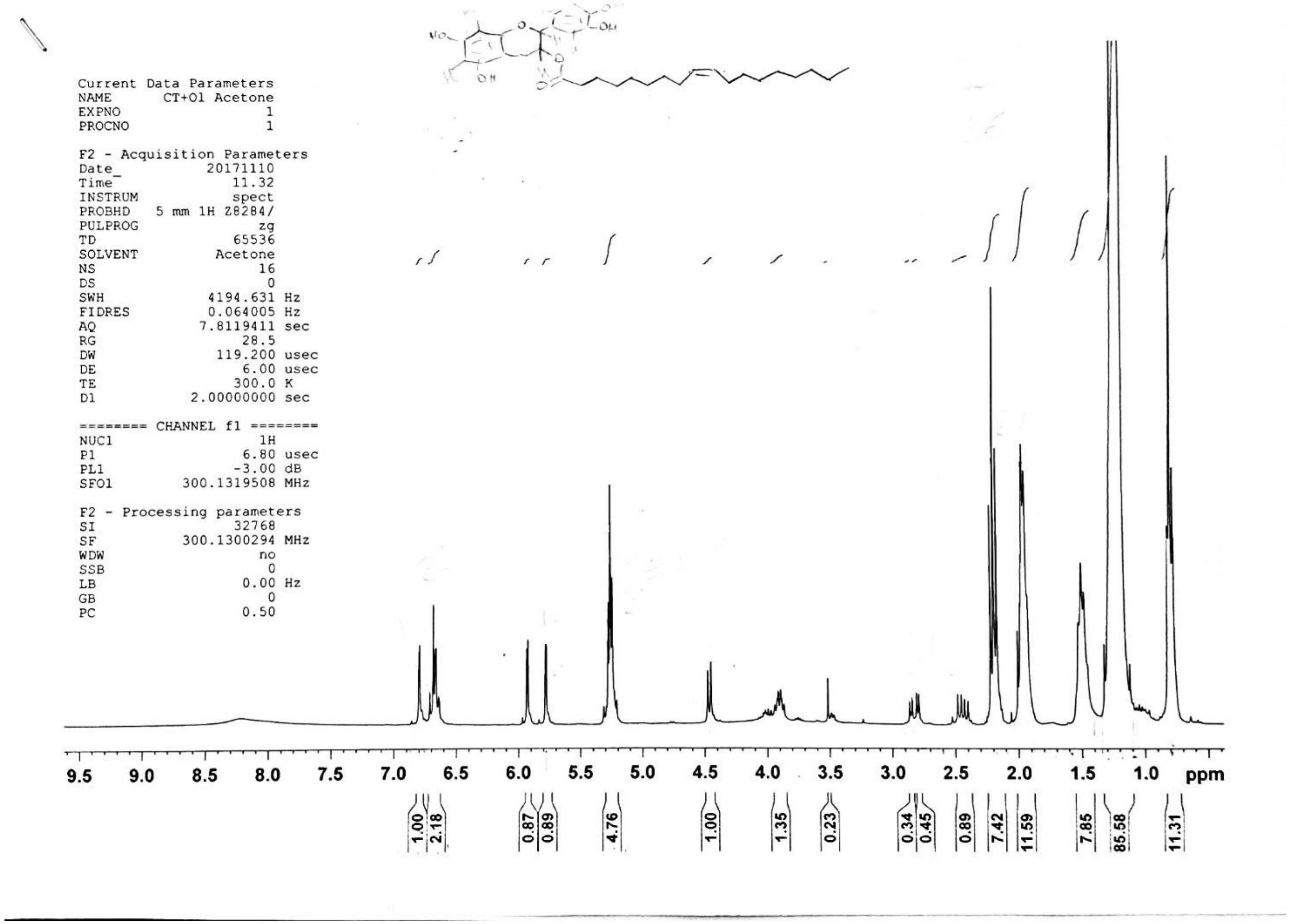

Figure S8. ^{13}C NMR of compound **5**

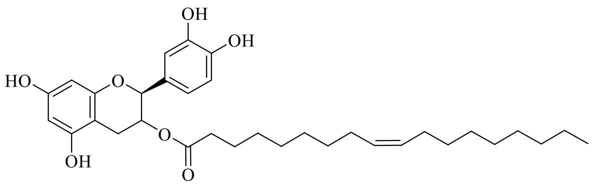

5

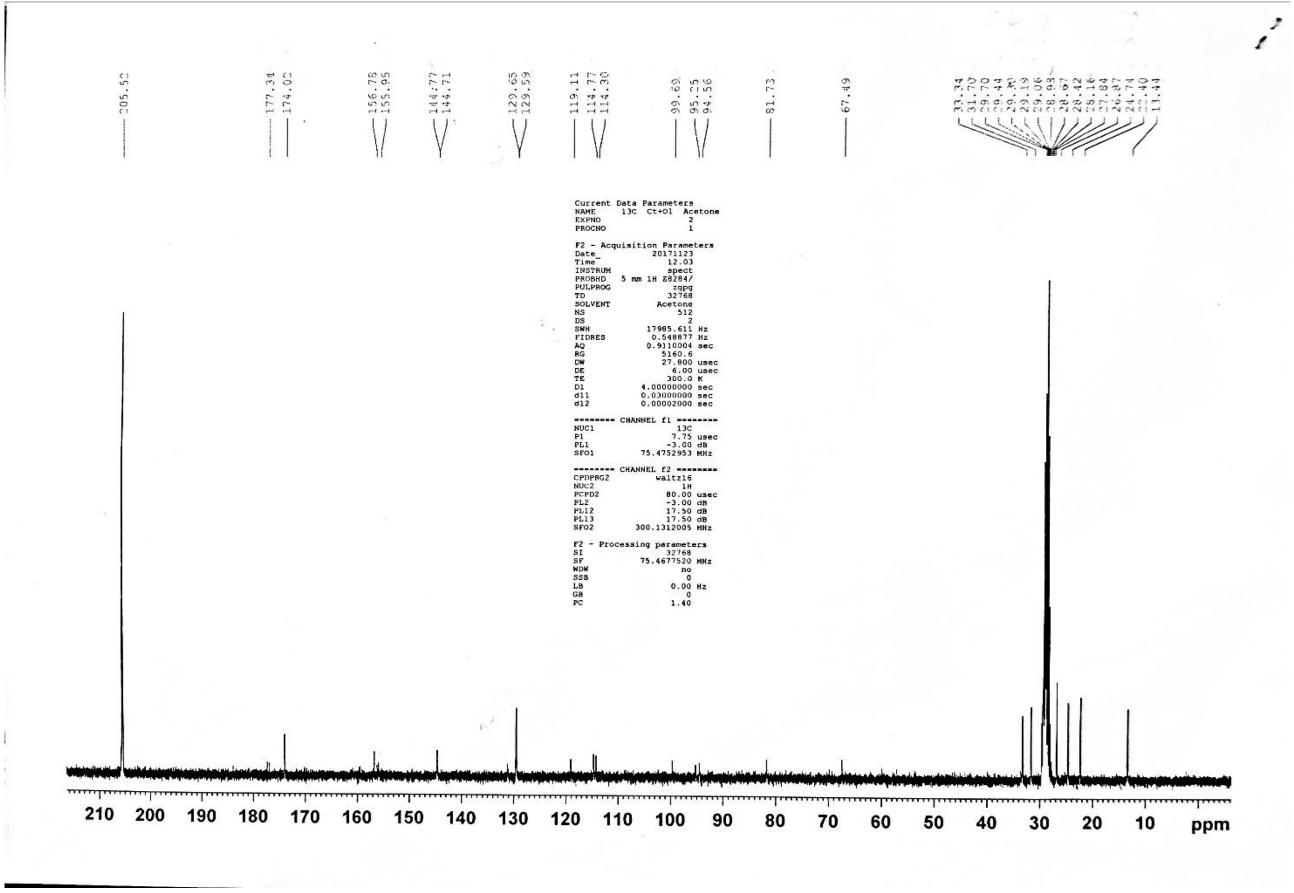

Figure S9. ^{1}H NMR of compound **6**

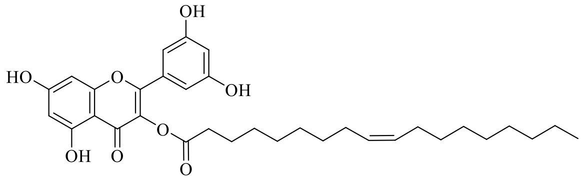

6

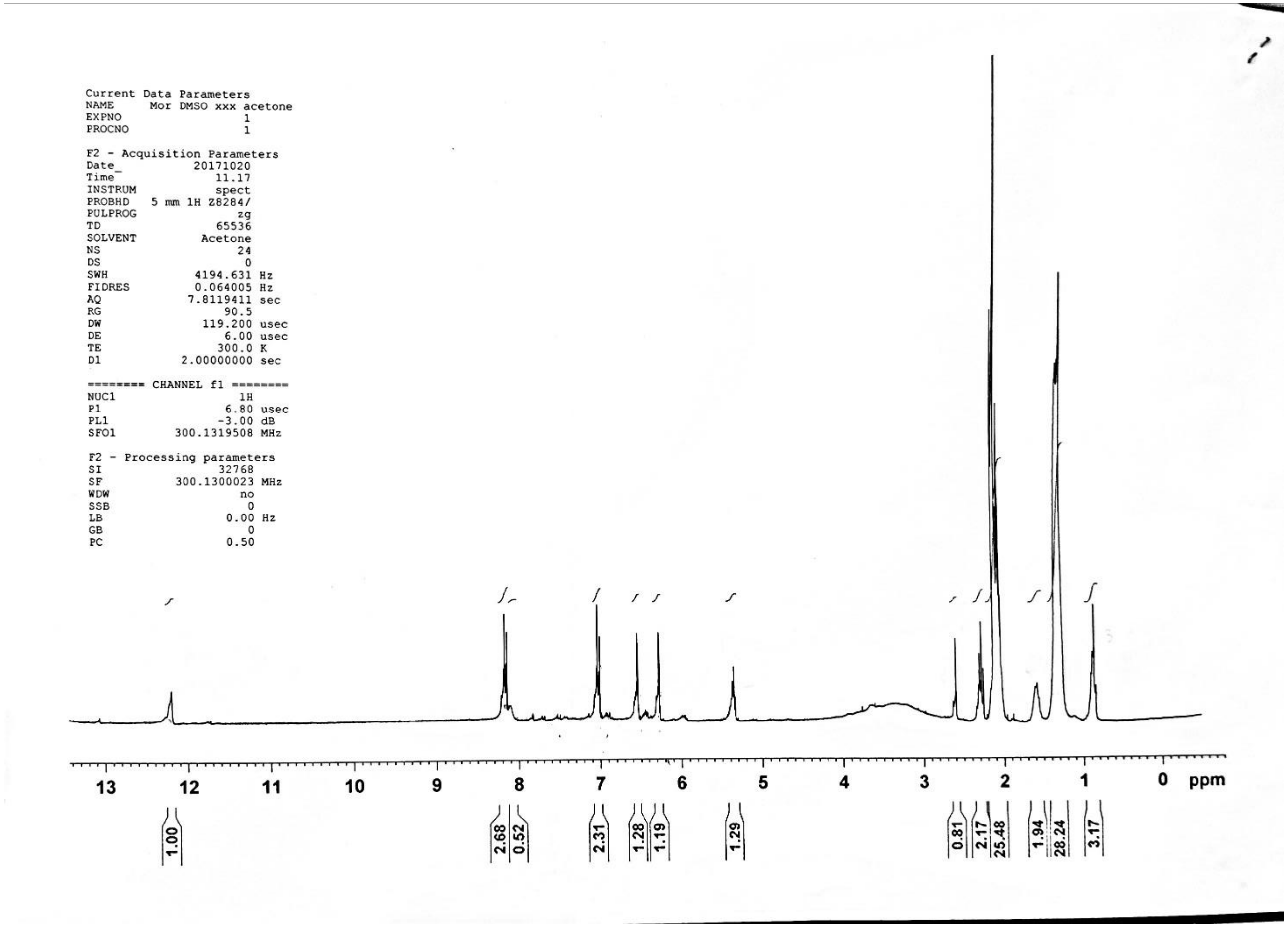

Supplement: Supplementary file 1 [file antioxidants-09-01077-s001.pdf]
